# Unearthing soil arthropod diversity through DNA metabarcoding

**DOI:** 10.7717/peerj.12845

**Published:** 2022-02-01

**Authors:** Monica R. Young, Paul D. N. Hebert

**Affiliations:** 1 Centre for Biodiversity Genomics, University of Guelph, Guelph, Ontario, Canada; 2 Department of Integrative Biology, University of Guelph, Guelph, Ontario, Canada

**Keywords:** Metabarcoding, Arthropods, Soil, Biodiversity, NGS, Environmental DNA

## Abstract

DNA metabarcoding has the potential to greatly advance understanding of soil biodiversity, but this approach has seen limited application for the most abundant and species-rich group of soil fauna–the arthropods. This study begins to address this gap by comparing information on species composition recovered from metabarcoding two types of bulk samples (specimens, soil) from a temperate zone site and from bulk soil samples collected at eight sites in the Arctic. Analysis of 22 samples (3 specimen, 19 soil) revealed 410 arthropod OTUs belonging to 112 families, 25 orders, and nine classes. Studies at the temperate zone site revealed little overlap in species composition between soil and specimen samples, but more overlap at higher taxonomic levels (families, orders) and congruent patterns of *α*- and *β*-diversity. Expansion of soil analyses to the Arctic revealed locally rich, highly dissimilar, and spatially structured assemblages compatible with dispersal limited and environmentally driven assembly. The current study demonstrates that DNA metabarcoding of bulk soil enables rapid, large-scale assessments of soil arthropod diversity. However, deep sequence coverage is required to adequately capture the species present in these samples, and expansion of the DNA barcode reference library is necessary to improve taxonomic resolution of the sequences recovered through this approach.

## Introduction

The “enigma of soil animal species diversity” (Anderson, 1975) will only be resolved through taxonomically comprehensive, large-scale surveys (Cameron et al., 2018; Decaëns, 2008). Although arthropods likely comprise the largest fraction of soil fauna diversity (Geisen et al., 2019), their taxonomy is so poorly understood that estimates of their species richness span two orders of magnitude (André, Noti & Lebrun, 1994; Erwin, 1982; Stork, 1988). Detailed assessments of soil biodiversity have been constrained by several factors, but inefficient methods for isolating arthropods from the soil matrix is an initial barrier (André, Ducarme & Lebrun, 2002). Once specimens are isolated, their high abundance combined with cryptic morphology, lack of taxonomic expertise, and the high incidence of undescribed species pose serious challenges to their identification (André et al., 2001; Bickford et al., 2007). Consequently, past studies of the soil fauna have typically examined a few taxonomic groups on a local scale or have employed coarse taxonomic resolution at a larger scale (Donoso, Johnston & Kaspari, 2010; Marra & Edmonds, 2005). However, these barriers can be resolved by DNA-based approaches because most species can be distinguished by examining sequence variation in the 648 bp barcode region of the mitochondrial cytochrome *c* oxidase I (COI) gene (Hebert et al., 2003; Hendrich et al., 2015; Zahiri et al., 2017). Moreover, specimens can be assigned to operational taxonomic units (OTUs), an effective proxy for species, enabling large-scale, comprehensive assessments of diverse arthropod assemblages (Hebert et al., 2016).

Although DNA barcodes reliably discriminate species of soil organisms (Hogg & Hebert, 2004; Raupach et al., 2016; Schäffer, Kerschbaumer & Koblmüller, 2019), this approach is time consuming and expensive as it involves processing individual specimens (Borisenko, Sones & Hebert, 2009). Metabarcoding, the coupling of DNA barcoding with high-throughput sequencing, enables time- and cost-effective assessments of diversity by allowing the analysis of bulk samples of specimens or soil (Taberlet et al., 2012). Until recently, metabarcoding has seen limited application for the characterization of soil arthropod biodiversity (Arribas et al., 2016; Dopheide et al., 2019a; Oliverio et al., 2018). The results from such assessments are likely to differ from those based on morphological study because reference libraries are incomplete and PCR amplification introduces bias (Schenekar et al., 2020). Because methods used to extract arthropods from soil are inefficient, direct analysis of DNA extracted from soil may permit more comprehensive species recovery (Deiner et al., 2017), but ‘relic’ DNA in soils may overestimate biodiversity and dampen ecological signals by conflating contemporary and historic species assemblages (Nagler et al., 2018). Despite these complexities, a comparison of metabarcode data from soil and soil-isolated specimens revealed similar patterns of arthropod *α*- and *β*-diversity although the two sampling methods recovered complementary fractions of the biota (Oliverio et al., 2018). However, no other studies have previously evaluated congruence in *β*-diversity patterns of soil arthropod communities using these methods.

This study begins by comparing patterns of *α*- and *β*-diversity at a nature reserve in southwestern Ontario revealed by DNA metabarcoding paired samples, one based on DNA extracted from the soil and a second based on DNA from specimens isolated from the soil. The recovery of OTUs, families, and orders was compared between the two methods. As well, results were validated against family- and order-level identifications based on morphological examination of specimens recovered after DNA extraction. The study then considers diversity patterns at a larger geographic scale by extending analysis to include soil samples from eight sites in Arctic Canada. While additional invertebrate and microbial taxa were recovered at all locales, analyses focused on arthropods, especially mites.

## Methods

### Sample collection

**Figure 1:**
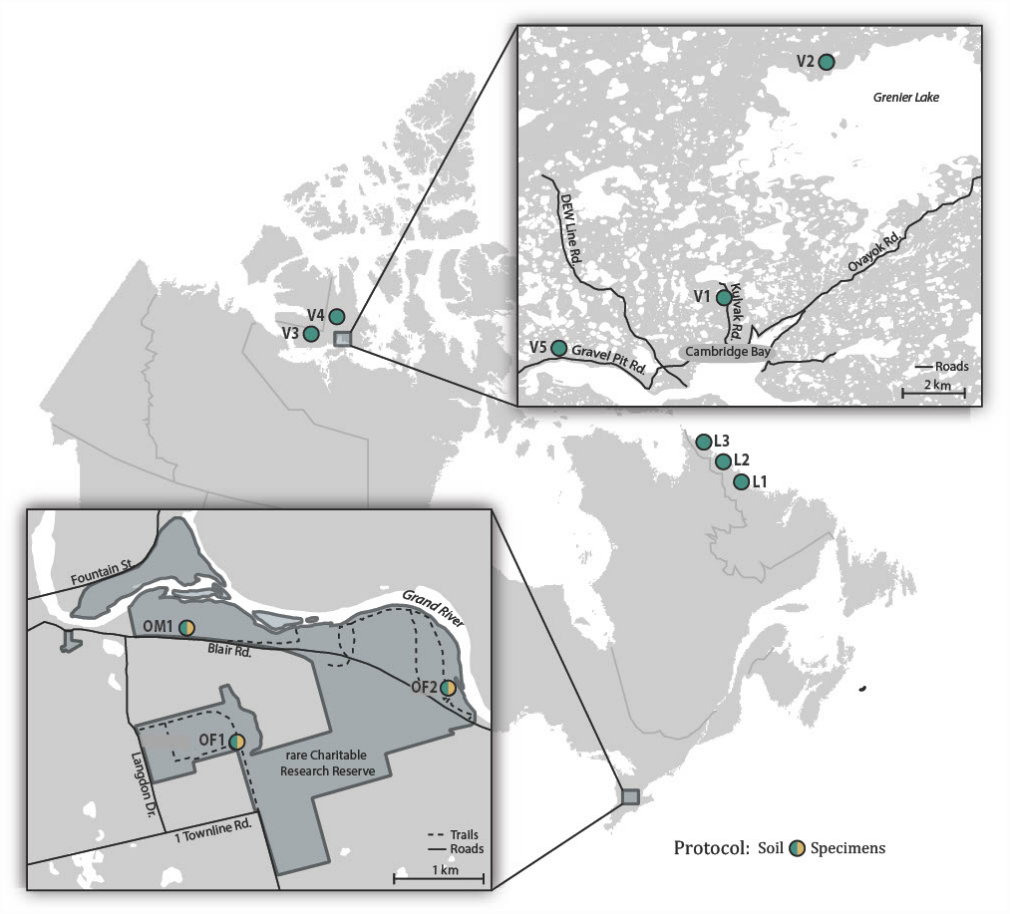
Map showing the 11 Canadian sites where soil samples were collected and the three sites where specimen samples were also obtained.

Soil samples were collected in July 2017 from two forested sites (OF1, OF2) and one meadow site (OM1) in ***rare***, a research reserve in the Eastern Temperate Forests ecoregion (EPA, 2015) in Ontario, Canada (Fig. 1; Table S1). Sampling at each of these sites involved collecting approximately 100 g of soil and the surface leaf litter at 3 m intervals along a 30 m transect. The 10 subsamples from each site were homogenized and pooled using a litter sifter. The sifter and tools used to collect each sample were sterilized between sites using H_2_O to remove residual debris followed by soaking in 1:10 diluted bleach for 15 min, a final rinse with H_2_O, and were then air dried. Approximately a tenth (100 g) of the total soil sample from each site was retained for bulk soil analysis, while the remaining ∼900 g of soil was placed in a modified Berlese-Tullgren funnel for one week with the isolated invertebrates preserved in 95% EtOH. We subsequently refer to these as “soil” and “specimen” samples. All samples were stored at −20 °C prior to DNA extraction.

To obtain comparative data from distant locales, samples were collected in July 2017 at three sites (L1-L3) spanning the transition from the Taiga to Arctic Cordillera ecoregions in coastal Labrador, and in July–August 2018 at five sites (V1-V5) in the Tundra ecoregion on southern Victoria Island near Ikaluktutiak (Cambridge Bay), Nunavut (Fig. 1; Table S1). At each site, soil samples were collected along two parallel 30 m transects (approximately 10 m apart) producing 16 samples that were processed using the same protocol as for the ***rare*** soil samples.

Samples were collected with permission at rare Charitable Research Reserve. Samples collected in the vicinity of Cambridge Bay fall under a Department of Environment Wildlife Research Permit (WL 2018-044). A permit was also obtained through the Canada C3 Expedition for sampling in National Parks in Labrador (ANP-2017-25145), while collecting in other sites was approved by the Nunatsiavut Government Research Advisory Committee.

### Positive control

A DNA extract prepared from a bulk arthropod sample was used as a positive control (CCDB positive control #07682). To gain a detailed understanding of its Barcode Index Number (BIN) diversity (Ratnasingham & Hebert, 2013), each of the component specimens (2,018 insects, 17 arachnids) were individually DNA barcoded. These specimens derived from an 8-day Malaise trap deployment at the Arkell Research Farm near Guelph, Ontario. DNA was extracted from a single leg of each specimen and the 658 bp barcode region was recovered using standard protocols (Ivanova, de Waard & Hebert, 2006; Hebert et al., 2013) utilizing the C_LepFolF/C_LepFolR primer pair (Hernández-Triana et al., 2014). When these primers failed to generate a sequence, the DNA extract was amplified using the MLepF1/C_LepFolR and C_LepR1/MLepR2 primer pairs (Hebert et al., 2013) followed by NGS failure tracking (Prosser et al., 2016) and SMRT sequencing (Hebert et al., 2018) when necessary. All sequences were uploaded to the public AGAKS project on BOLD, the Barcode of Life Dataystem (Ratnasingham & Hebert, 2007). Those meeting quality standards (≥ 500 bp, <1% ambiguous bases, free of contamination and stop codons) were assigned a BIN. In total, 432 BINs from 11 orders and 90 families were represented among the 1,917 sequences that gained an assignment. The other 112 specimens failed to generate a quality sequence and consequently lacked a BIN assignment. Some of these specimens (1 Coleoptera, 36 Diptera, 5 Hemiptera, 75 Hymenoptera, 1 Lepidoptera) may represent BINs that were not characterized.

The bulk sample was then reconstituted by pooling all specimens and a bulk DNA extract was made following standard protocols (Braukmann et al., 2019) for use as a positive control in subsequent metabarcoding analyses.

### Molecular analysis of specimen and soil samples

DNA was extracted from the bulk specimen samples using a non-destructive protocol so that the exoskeletons of the specimens were available for morphological study after lysis. Extraction began with the filtration of each sample through a sterile Microfunnel (0.2 µm Supor Membrane Filter, Pall Laboratory) followed by the addition of 10 mL of insect lysis buffer (Ivanova, de Waard & Hebert, 2006). The lysate was mixed gently and incubated overnight at 56 °C, and eight 50 µl aliquots were then transferred from each lysate into separate wells in a 96-well microplate to create technical replicates. Specimen exoskeletons were recovered from the remaining ∼9.5 mL of lysate through filtration and stored at room temperature in 95% EtOH. 100 µl of binding mix was added to each well of the microplate and the resultant 150 µl solution was transferred to a 3.0 µm glass fibre (GF) plate (Pall Laboratory) followed by centrifugation at 5,000 g for 5 min. 180 µl of protein wash buffer was then added to each well in the plate and centrifuged at 5,000 g for 5 min. This step was followed by two additional rounds of wash buffer (600 µl each) and centrifugation for 5 min at 5,000 g. The GF plates were incubated for 30 min at 56 °C to evaporate residual EtOH before DNA was eluted into clean 96-well Eppendorf plates by adding 50 µl of 10 mM Tris–HCl (pH 8.0) and centrifugation at 5,000 g for 5 min.

DNA was extracted from the bulk soil samples using a slightly modified version of a standard protocol (Ivanova, Fazekas & Hebert, 2008). The ∼100 g aliquot of soil from each sample was mixed with 200 mL of insect lysis buffer which included 1% polyvinylpyrrolidone (PVP). The resulting lysates were mixed gently on a shaker and incubated overnight at 56 °C. Technical replicates were constructed by transferring eight 50 µl subsamples per lysate into a 96-well microplate. Negative extraction controls were also created by adding four 50 µl aliquots of ddH_2_O to each 96-well plate. Each lysate (and control) was mixed with 100 µl of plant binding buffer, transferred to a 3.0 µm GF plate, and centrifuged at 5,000 g for 5 min. Each well in the GF plate was then washed with 150 µl of the plant binding buffer and centrifuged for 2 min at 5,000 g, followed by 150 µl of binding mix centrifuged at 5,000 g for 2 min, and two rounds of wash buffer (750 µl each) centrifuged for 5 min at 5,000 g. The GF plates were incubated for 30 min at 56 °C before DNA was eluted into clean 96-well Eppendorf plates with 50 µl of 10 mM Tris–HCl pH 8.0 centrifuged at 5,000 g for 5 min.

In total, three 96-well DNA plates were used to accommodate the 176 technical replicates (22 samples × 8 replicates each; Table S2). Each plate included up to 80 technical replicates (10 samples × 8 replicates each) as four wells were treated as negative extraction controls and 12 wells were left empty for PCR controls (8 positive, 4 negative) that were added later. Samples in each plate were grouped by ecoregion. Although DNA from the paired specimen/soil samples was extracted separately (as they required different protocols), they were transferred into the same plate following extraction. The first plate included all 48 replicates from the six Eastern Temperate Forests samples; the second included the six Taiga/Arctic Cordillera samples (48 replicates); and the third included all ten Tundra samples (80 replicates). Although the number of replicates differed by plate, there was no significant difference in their number of post-filtered reads (ANOVA *F*_2,173_ = 2.2, *p* = 0.2).

DNA extracts were stored at 4 °C for ≤4 days prior to PCR. Immediately before analysis, 50 µl of template DNA from the positive control sample was added to each of the eight wells in Row 11 while wells in Row 12 were employed as negative controls. Amplicon libraries were then constructed using a two-stage PCR protocol (Braukmann et al., 2019) that employed the AncientLepF3 (Prosser et al., 2016) and C_LepFolR primers to amplify a 463 bp fragment of COI and attach unique molecular identifiers (UMIs) with Ion Torrent S5 sequencing adaptors. The labelled products from each plate were pooled and normalized to 1 ng/µl prior to sequencing. Sequencing libraries were prepared on the Ion Chef™ (Thermo Fisher Scientific) following manufacturer’s instructions and were then analyzed on an Ion Torrent S5 using a 530 chip.

All molecular analysis was performed at the Canadian Centre for DNA Barcoding (CCDB; http://www.ccdb.ca). The DNA extracts were archived at −80 °C at the CCDB while the vouchers were deposited in the specimen archive at the Centre for Biodiversity Genomics.

### Sequence analysis

The raw sequence data was analyzed with the ‘JAMP’ (Elbrecht, 2018) package in R which utilizes functions from USEARCH (Edgar, 2013), VSEARCH (Rognes et al., 2016), and Cutadapt (Martin, 2011). The forward primer was first trimmed from each read and the resultant sequence was truncated to 463 bp. Reads shorter than 350 bp in length or with more than 0.5% expected errors (*E*max = 2.3) were discarded. *E* is the sum of error probabilities based upon the quality (phred) score for each nucleotide position (Edgar & Flyvbjerg, 2015). Adopting an *E* max of 2.3 (= error rate of 0.5%) enabled a balance between read retention and error filtering since most reads (98%) were excluded by a strict error threshold (*E*max = 1) while a higher threshold (*E*max = 4.6; error rate = 1%) did not improve the recovery of known BINs in the positive control despite the recovery of more OTUs. The remaining reads were dereplicated with simultaneous chimera filtering and those with more than one replicate were clustered into OTUs at 3% nucleotide divergence using UPARSE (Edgar, 2013) as implemented by USEARCH in JAMP. Singleton reads were assigned to existing OTUs if their divergence was <3% to a cluster; otherwise they were discarded. OTUs with low abundance in a replicate (<0.05%) were also excluded (Table S3). The maximum read count for any OTU in the negative controls was subtracted from all other occurrences of that OTU to minimize the potential effects of tag switching and background contamination. Consequently, nine OTU occurrences were reduced to zero and one OTU was filtered entirely (Table S4). Although amino acid translation-based filtering to remove reads with stop codons and frameshift indels (1–2 bp insertions/deletions) can reduce the prevalence of spurious OTUs, this procedure is rarely adopted since it is not easily replicable using standard bioinformatics tools (Creedy et al., 2021). Its use is further complicated when samples contain a broad range of taxonomic groups as their analysis requires the use of several translation tables. Consequently, OTUs with stop codons and frameshift indels were not filtered from our dataset.

OTUs were assigned the taxonomy of their top hit in the reference library [June 2021] on BOLD (Ratnasingham & Hebert, 2007) using BOLDigger (Buchner & Leese, 2020) in Python v3.9 (Python Software Foundation). JAMP’s default parameters for taxon assignment at the species (≤2% divergence), genus (≤5%), family (≤10%), and order (≤15%) levels were adopted for all OTUs except those with top hits to Acari (mite) orders where lineage-specific thresholds for family and order identification were employed (P_95_; Young, de Waard & Hebert, 2021). To assess the extent of species-level library coverage, OTUs ≤2% divergent from a reference sequence in BOLD were categorized as ‘known’ while OTUs with higher divergence were categorized as ‘novel’.

OTUs in the positive control were compared to the BINs detected through detailed DNA barcode analysis of its component specimens to evaluate recovery of the positive control. In cases where multiple OTUs matched to the same BIN, their consensus sequences were examined for stop codons and frameshifts. Such features may reflect amplification or sequencing errors but could also indicate the recovery of nuclear mitochondrial pseudogenes (NUMTs). OTUs that did not closely match a BIN known to be present in the positive control were also scanned for possible signs of NUMT recovery (*e.g.*, stop codons and frameshifts), and their read counts were compared with the experimental samples to assess possible cases of tag-switching.

### Specimen vs soil analysis

Taxon recovery, composition, and diversity patterns were compared for the paired specimen and soil samples from Ontario. First, OTU accumulation curves (with 1,000 replicates) were generated using the BiodiversityR package (Kindt & Coe, 2005) in R version 4.0.2 (R Core Team, 2020) for all taxa combined and for arthropods separately to assess similarity among the technical replicates for each sample. The technical replicates for a sample were then pooled, and variation in taxon richness was evaluated at three levels (order, family, OTU) for all taxa combined, and for all arthropods and mites separately. One-tailed paired t-tests were used to determine if richness was lower for specimen than for soil samples. In cases where the assumption of normality was not met, one-sided Wilcoxon Sign Rank tests (SRT) were used. Since significant differences were found for all taxa combined but not for arthropods or mites, variation was also assessed for non-arthropod invertebrates and protists as potential explanatory factors. Bacteria, fungi, and chordates were excluded from these analyses since they comprised a very small fraction of the total richness.

Variation in the recovery of arthropod orders and families was compared with the morphologically identified specimens from the Berlese-Tullgren samples (Table S5). Patterns of arthropod and mite *β*-diversity were evaluated through the incidence-based Sorensen dissimilarity metric estimated for each pair of samples using the betapart (Baselga & Orme, 2012) package in R. Dissimilarity values range from 0 to 1, with 0 indicating complete OTU overlap, and 1 indicating no shared OTUs. Compositional differences between the two methods were visualized from Sorensen distances using complete-linkage hierarchical clustering in R, and tested for significance through permutational multivariate analysis of variance (perMANOVA; 1,000 randomizations) with the vegan package (Oksanen et al., 2015) in R.

### Diversity patterns of soil arthropod assemblages

The soil samples from the eight Arctic sites were analyzed in a similar fashion to those from ***rare***. Accumulation curves were first used to assess similarity among the technical replicates for each sample and replicates were then pooled by sample. Patterns of arthropod and mite *β*-diversity were then evaluated with Sorensen’s dissimilarity metric. Mantel test correlations (1,000 randomizations) of Sorensen dissimilarity and geographic distance matrices were used to estimate the proportion of variation in *β*-diversity explained by spatial structure through the ade4 package in R (Dray & Dufour, 2007). Compositional differences between samples, sites, and ecoregions were visualized through complete-linkage hierarchical clustering of Sorensen dissimilarities, and tested for significance with spatial scale (Site, Ecoregion) as explanatory factors. The contributions to overall diversity made at each spatial scale were evaluated by hierarchically partitioning the total arthropod and mite OTU richness (*γ*-diversity) into its sample, site, and ecoregion components using PARTITION (Veech & Crist, 2009). Both additive (*γ* = *α*Sample + *β*Sample + *β*Site + *β*Ecoregion) and multiplicative (*γ* = *α*Sample × *β*Sample × *β*Site × *β*Ecoregion) partitions were calculated to assess net (*i.e.*, *β*Site = *α*Ecoregion − *α*Site) and proportionate (*i.e.*, *β*Site = *α*Ecoregion/ *α*Site) increases in OTU richness at successive spatial scales. To test whether the partitions differed significantly from chance they were compared to null models using PARTITION’s restricted individual-based randomization following Crist et al. (2003).

## Results

### Controls

In total, 142 reads representing 109 OTUs were recovered from the 24 negative controls. The maximum read abundance (≤6) for each of these OTUs was subtracted from its occurrence in each replicate during the final filtering step, causing one OTU to be eliminated. All other OTUs had higher read counts in the positive control or specimen/soil samples (29–300K reads) compared to the negative controls (1–6 reads), and most (96.2%) had >100 reads in another sample indicating that their presence in the negative controls likely reflected tag-switching.

After filtering, 0.5M reads representing 161 OTUs were recovered from the 24 positive controls (Table 1) with an average of 141 OTUs (SD = 9.5) in the 8 replicates included in each run. Five of these OTUs (4 arthropods, 1 possible heterokont) reflected tag switching as they had low read counts in the positive controls (7–71 reads) versus >100K reads in the specimen/soil samples. Three quarters of the other OTUs (115/156) were close matches (≤2% divergence) to a BIN known to be present in the positive control, meaning that 27% of the 432 BINs in the positive control were recovered across the three runs. Within single runs, the recovery of BINs in the positive control averaged 24% (= $\bar {x}$ = 104 OTUs, SD = 7.6). Some of the other 41 OTUs were clearly NUMTs derived from a species in the control sample. For example, one OTU was a perfect match to a dipteran BIN (BOLD:AAU6656; *Neolimnophila pacida*) in the positive control, while a second OTU with 6.8% divergence possessed a stop codon and 5 bp deletion indicating that it was a NUMT. Six BINs abundant in the positive control were linked to several closely matching (≥95% similarity) OTUs that were clearly NUMTs. For example one OTU lacking stop codons and frameshifts matched 100% to a fly BIN (BOLD:AAH3914; *Sciara humeralis*) that was represented by 75 specimens in the positive control. Two more OTUs matched this BIN but both possessed a frameshift indel; one was a perfect match bar the indel while the other was 2.2% divergent. Three other OTUs showed a close match (≥98.9% similarity) to the sole orthopteran BIN (BOLD:AAA4555; *Melanoplus*; 5 specimens) that represented the largest biomass of any species in the sample, but just one possessed a frameshift indel. A similar scenario was observed for six OTUs matching (≥92% similarity) an abundant fly BIN (BOLD:AAG6745; *Pollenia*; 32 specimens) where just one of the six OTUs possessed a frameshift indel. Such cases likely indicate the detection of young NUMTs, but might also reflect BINs present in the positive control that failed to sequence in the initial characterization of the sample. For example, 14 OTUs lacking stop codons and frameshift indels were unique to the positive controls and consistently recovered in each replicate. They represented a bacterium (*Wolbachia pipientis*) and 13 arthropods that might derive from gut contents or specimens in the positive control that lacked a BIN assignment.

**Table 1:** Summary of post-filtered reads and OTUs generated from metabarcoding 19 soil samples and three soil invertebrate samples from 11 Canadian sites. Data is summarized separately for the positive and negative controls, all experimental samples combined, the subset of paired specimen and soil samples, and the soil samples alone.

Source	Samples/ Replicates	Total Reads	Total OTUs	Mean Reads/ Replicate (SD)	Mean OTUs/ Replicate (SD)	Mean Reads/ Sample (SD)	Mean OTUs/ Sample	Unidentified reads	Unidentified OTUs	Identified Reads	Identified OTUs
Negative controls	- /24	142	109	6.5 (8.8)	5.9 (8.0)	–	–	75	65	67	44
Positive controls	- /24	544,897	161	22,704 (14,836)	141 (4.3)	–	–	7	1	544,890	160
All samples	22/176	3,726,640	7,487	21,295 (17,879)	106 (67)	169,393 (126,444)	514 (297)	2,214,161	6,336	1,512,479	1,151
Specimens + Soil	6/48	1,214,492	2,126	25,840 (20,422)	109 (97)	202,415 (173,734)	439 (456)	286,257	1,776	928,235	350
Specimens	3/24	812,090	120	33,837 (26,086)	28 (10)	270,697 (247,020)	47 (22)	798	37	811,292	83
Soil	3/24	402,402	2,052	17,496 (4,246)	193 (71)	134,134 (21,271)	831 (229)	285,459	1,764	116,943	288
All Soil	19/152	2,914,550	7,414	19,302 (15,404)	119 (64)	153,397 (99,461)	587 (246)	2,213,363	6,324	701,187	1,090

### Sequences from specimen and soil samples

**Figure 2:**
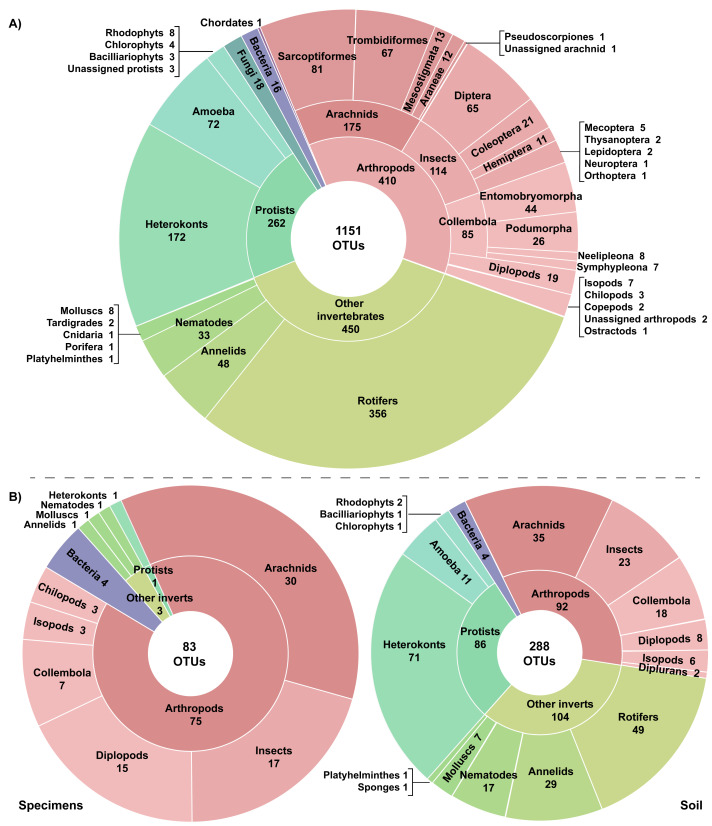
Taxonomic breakdown of the classified reads and OTUs generated from (A) 22 samples including three specimen and 19 soil samples, and (B) the subset of six paired specimen and soil samples.

A total of 27.75 million reads were recovered from the 22 experimental samples. Approximately 13% (3,726,640) of these reads were retained post-filtering with an average of 21.4K reads (SD = 14.8K) per replicate and 169.4K reads (SD = 126K) per sample (Table 1). These reads were assigned to 7,487 OTUs with an average of 106 OTUs (SD = 67) per replicate and 514 OTUs (SD = 297) per sample. Many OTUs (84.6%) recovered from the 22 samples showed low similarity to any sequence in the BOLD reference library (<64.3% to a Trombidiformes, <74.9% to a Sarcoptiformes, <75.3% to a Mesostigmata, <85.0% to all other taxa). As a result, 6,366 of the 7,487 OTUs failed to gain a taxonomic assignment (Table 1). Examination of the top hits for these unidentified OTUs revealed that nearly half (38.6%) were protists with heterokonts comprising more than half their total (20.1%). Many other unassigned OTUs were likely arthropods (19.0%), other invertebrates (22.3%), or bacteria (14.5%). However, 1,151 OTUs did gain a phylum or better assignment. They included representatives of 20 phyla, 30 classes, and 60 orders (Fig. 2). More than a third of the identified OTUs (35.6%) were arthropods; these 410 OTUs included representatives of 112 families, 25 orders, and nine classes. However, other invertebrate lineages were also well represented (39.1% of identified OTUs), while protists (22.8%), fungi (1.5%), and bacteria (1.0%) were also recovered. Another OTU showed highest similarity (87.5%) to a fish sequence but BLAST analysis indicated that it was a human COI NUMT. Two other identified OTUs (Cnidaria, Porifera) appeared to be spurious since they were detected in soil >2 km from water and their top matches on BOLD and GenBank included protists and fungi in addition to cnidarians and poriferans. Stop codons and frameshift indels were noted in about 20% of the identified OTUs, but most involved a single nucleotide indel in a poly-T tract or near the 3′-end, potentially reflecting NUMT recovery or PCR/sequencing errors.

**Figure 3:**
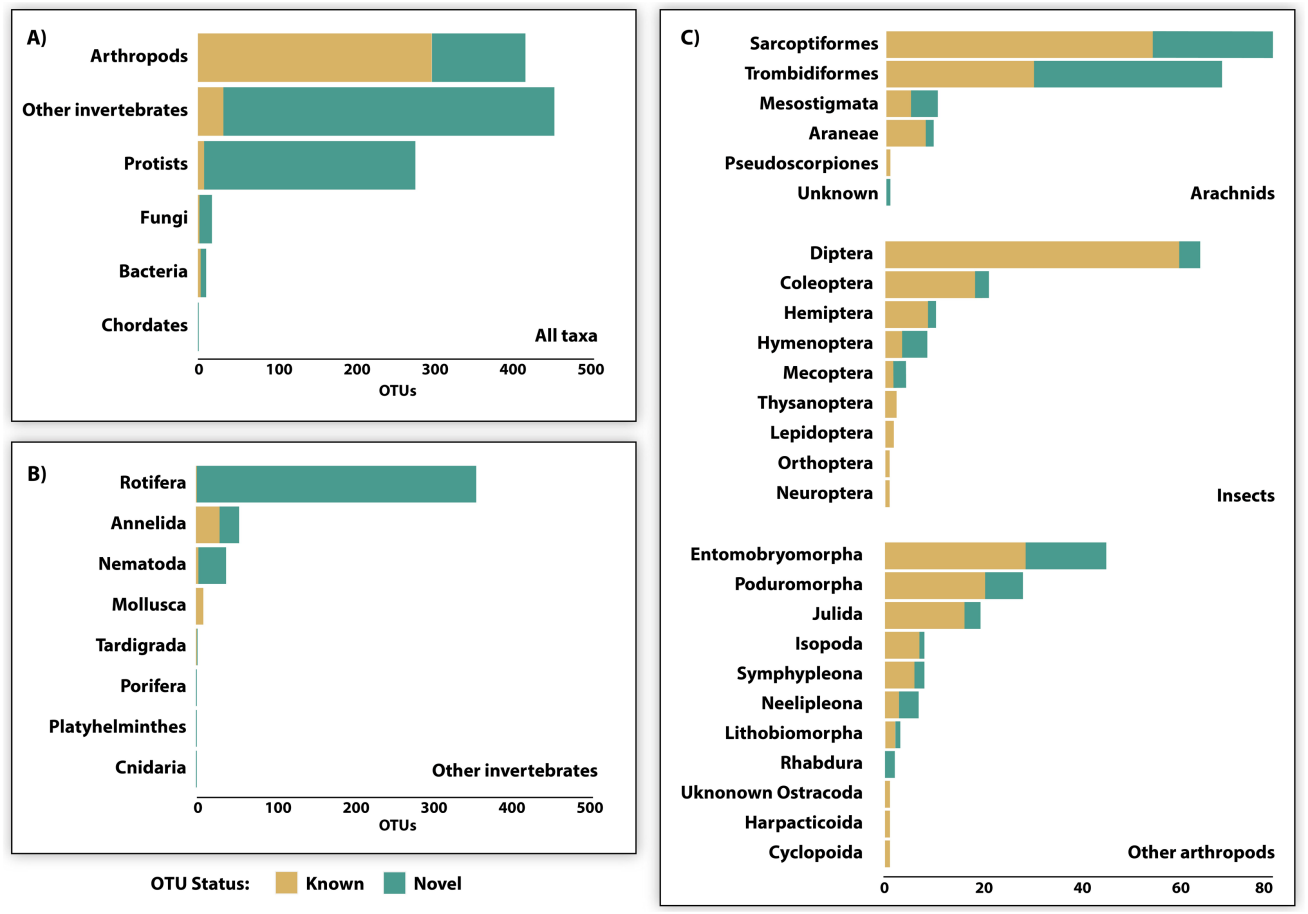
OTU diversity recovered by DNA metabarcoding 22 soil samples (3 specimen, 19 soil) and the proportion of known and novel OTUs based on comparison with the BOLD reference library. Comparisons are provided for (A) 6 major taxonomic groups, (B) 8 invertebrate phyla, and (C) 23 arthropod orders.

Just 28.6% of the identified OTUs were represented in the reference library on BOLD, and only 15.1% were assigned to a species (Fig. 3, Table 2). Because taxonomic coverage and parameterization of the barcode reference library varied among groups, identification success differed among taxa. For example, 67.1% of the arthropod OTUs matched (≥98% similarity) a reference sequence on BOLD and nearly a third (32.9%) were assigned a species. By contrast, just 8.9% of the OTUs in other invertebrate phyla matched a reference sequence and only 6.7% were identified to a species. Among arthropods, OTU matches on BOLD were considerably higher for insects (87.7%) and for other arthropods (60.1%) than for arachnids (54.3%). The low success for arachnids reflected the fact that just 47.9% of acarine OTUs found a match versus 83.3% for spiders (Fig. 3). While OTUs from some other invertebrate phyla (*e.g.*, Rotifera) also had very poor coverage in BOLD, all eight gastropod (Mollusca) OTUs and 56.2% of the Clitellata (Annelida) OTUs matched a reference sequence. By contrast, just 1 of 356 rotifer (Bdelloidea) OTUs matched a reference sequence and coverage was only slightly better for nematodes (Chromadoria, Enoplea; 8.0%). Parameterization improved at higher taxonomic levels, with 23.8% of all OTUs assigned to a genus, 44.0% to a family, 80.0% to an order, and 98.0% to a class (Table 2). Across all taxonomic levels, the proportion of OTUs gaining an identification was consistently higher for arthropods than for other phyla.

**Table 2:** The number of OTUs (and percent of total OTUs) assigned taxonomy at five major ranks based on comparison with BOLDs reference library. Data is summarised for all taxa combined and each major group detected from metabarcoding 19 soil samples and three soil invertebrate samples from 11 Canadian sites.

Taxon	Phylum	Class	Order	Family	Genus	Species
Arthropods	410 (100%)	408 (99.5%)	407 (99.3%)	382 (93.2%)	217 (52.9%)	135 (32.9%)
Other invertebrates	450 (100%)	448 (99.6%)	364 (80.9%)	96 (21.3%)	42 (9.3%)	30 (6.7%)
Protists	262 (100%)	257 (98.1%)	136 (51.9%)	26 (9.9%)	13 (5.0%)	7 (2.7%)
Fungi	17 (100%)	6 (35.3%)	6 (35.3%)	1 (5.9%)	1 (5.9%)	1 (5.9%)
Bacteria	11 (100%)	9 (81.8%)	7 (63.6%)	1 (9.1%)	1 (9.1%)	1 (9.1%)
Chordates	1 (100%)	1 (100%)	1 (100%)	0 (0%)	0 (0%)	0 (0%)
Total	1,151 (100%)	1,129 (98.0%)	921 (80.0%)	506 (44.0%)	274 (23.8%)	174 (15.1%)

### Specimens vs soil

**Figure 4:**
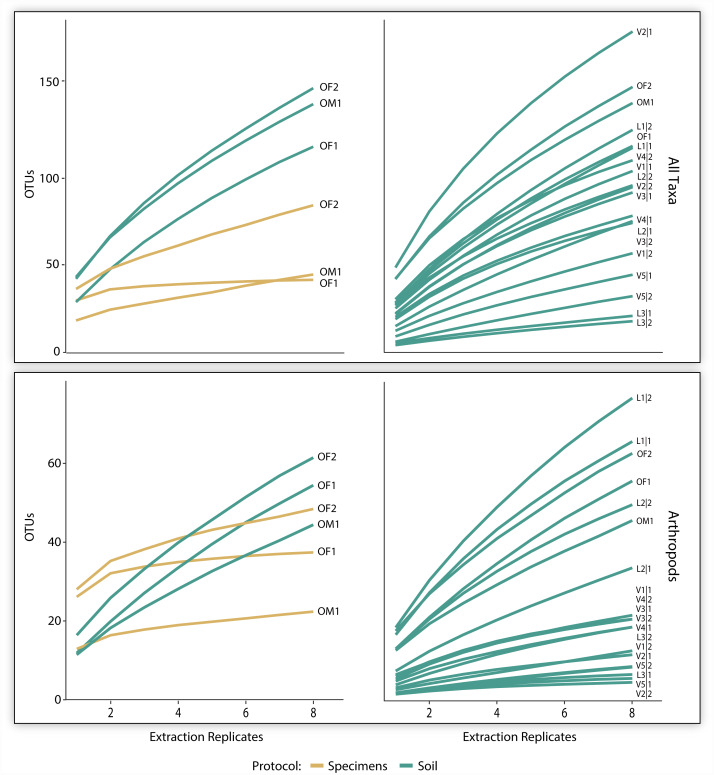
OTU accumulation curves (with 1,000 randomizations) across eight extraction replicates summarized for all taxa combined and all arthropods detected for the three paired specimen and soil samples, and all 19 soil samples.

The three pairs of samples included 2,126 OTUs and 1,214,492 reads, but the OTU count was 17x higher for soil than specimen samples (Table 1). Many of these OTUs (83.5%) could not be assigned taxonomy, even at a kingdom level. Among these unidentified OTUs, 99.3% derived from the soil versus 2.1% from the specimen samples (Table 1). Among the 350 OTUs that gained a taxonomic assignment, there were representatives of 76 families and 40 orders (Fig. 2). Nearly half (150) the identified OTUs were arthropods belonging to 62 families and 21 orders. OTU accumulation curves for the eight technical replicates indicated that only one sample reached an asymptote (OF1) and that the slope of the curves was consistently steeper for soil than specimen samples (Fig. 4). While a similar pattern was observed for the accumulation of arthropod OTUs, the slope of these curves was less than those for all taxa.

Few OTUs were shared between the specimen and soil samples from a particular site (Fig. S1). On average, 146 identified OTUs (SD = 12) were recovered from a site but just 7 (SD = 303) were shared between paired samples. The same trend was observed for arthropods and mites as mean site totals were 60 (SD = 9.5) and 23 (SD = 14) OTUs respectively with an average of just 5.7 (SD = 4.6) and 2.7 (SD = 2.9) OTUs shared. Many more orders, families, and OTUs were detected in the soil than specimen samples (Table 3) reflecting their much higher recovery of protozoan and non-arthropod invertebrate OTUs. Although soil samples recovered slightly more arthropods than specimen samples at all three levels in the taxonomic hierarchy, these differences were not significant (Table 3). There was also no significant difference between mite family and OTU richness with the two sampling approaches.

**Table 3:** Comparison of mean taxon richness detected in six paired specimen and soil samples for three levels of taxonomic resolution (order, family, OTU). Tests of significance are provided for differences in mean richness values by paired t-tests or, in cases with non-normal residual distributions, Wilcoxon Sign Rank tests (SRT).

		Specimens		Soil		
Taxon	Rank	Mean	(SD)	Mean	(SD)	Test of significance
All taxa	Order	9.3	(4.0)	27.3	(0.6)	2 = −8.6, *p* = 0.007
	Family	15.3	(5.5)	31.7	(5.1)	2 = −4.6, *p* = 0.02
	OTU	34.3	(14.2)	118.3	(18.9)	2 = −4.9, *p* = 0.02
Arthropods	Order	7.0	(2.6)	12.3	(0.6)	SRT *p* = 0.09
	Family	14.3	(5.5)	24.3	(3.7)	2 = −2.8, *p* = 0.05
	OTU	31.3	(12.4)	36.7	(6.5)	2 = −0.6, *p* = 0.3
Mites	Order	2.3	(0.6)	2.3	(0.6)	*NA*
	Family	7.3	(3.2)	9.0	(4.6)	2 = −1.8, *p* = 0.1
	OTU	13.0	(7.0)	13.0	(9.6)	2 = 0, *p* = 0.5
Other invertebrates	Order	1.5	(0.7)	9.0	(0.0)	2 = −3.6, *p* = 0.03
	Family	1.5	(0.7)	7.3	(1.5)	SRT *p* = 0.08
	OTU	1.5	(0.7)	40.7	(9.1)	2 = −7.3, *p* = 0.009
Protists	Order	0.3	(0.6)	5.7	(0.6)	2 = −3.6, *p* = 0.03
	Family	0.3	(0.6)	2.3	(0.6)	2 = −3.5, *p* = 0.04
	OTU	0.3	(0.6)	38.0	(6.9)	2 = −4.9, *p* = 0.02

**Figure 5:**
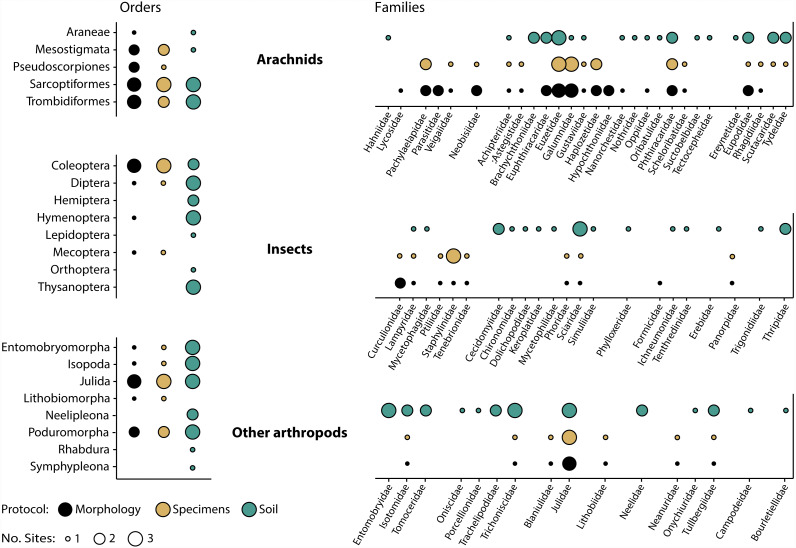
Taxonomic recovery of arthropod orders and families from three paired samples of specimens and soil analysed by DNA metabarcoding compared to taxa detected through morphological analysis of vouchers from the specimen samples.

Morphological inspection of specimens recovered after DNA extraction revealed that orders and families corresponded well with those detected by DNA analysis (Fig. 5). Just two (Araneae, Hymenoptera) of 14 orders and seven (Eupththiracaridae, Formicidae, Hypochthoniidae, Lycosidae, Nanorchestidae, Oppiidae, Parasitidae) of 35 families present among the morphologically identified taxa were not recovered by metabarcoding. Conversely, just two mite families (Trombidiformes: Scutacaridae, Tydeidae) detected by metabarcoding were not identified morphologically. These families were not present, even as immatures, as all 24 juvenile mites were Mesostigmata or Sarcoptiformes. Their detection could also not be explained by tag-switching or misidentification as they were unique to the sample and their sequence was deeply embedded within their family’s clade in the reference library. Compositional differences between the specimen and soil samples were also prominent as two orders and 16 families were detected by specimen but not by soil analysis, while seven orders and 32 families were recovered by soil but not by specimen analysis (Fig. 5).

**Figure 6:**
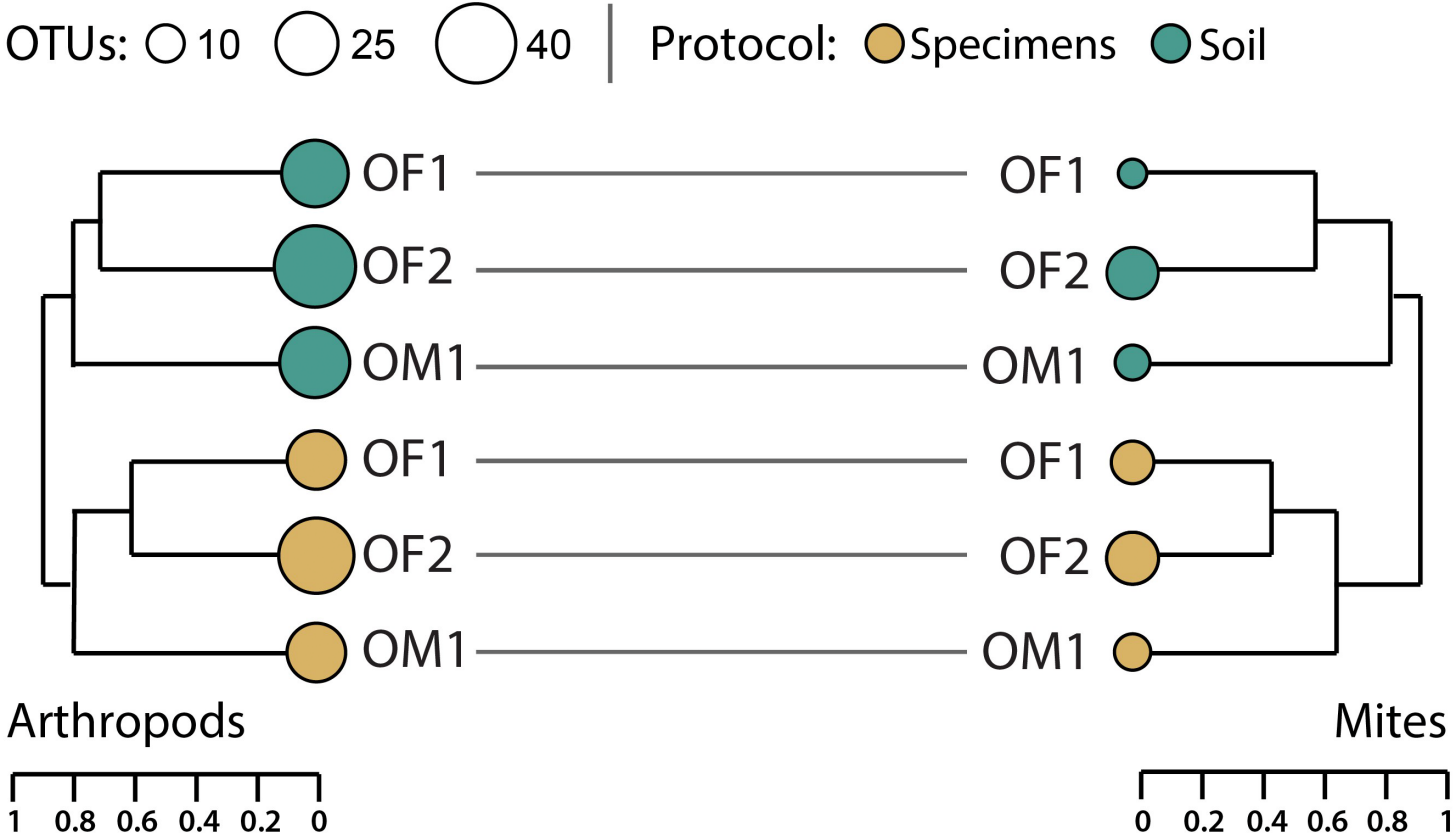
Hierarchical clustering of pairwise Sorensen dissimilarity values for arthropod and mite OTUs detected from three paired specimen and soil samples.

Hierarchical clustering of pairwise *β*-diversities suggested that compositional differences in arthropod OTU assemblages were greater between the paired soil/specimen samples than between sites although this difference was not significant (perMANOVA *R*^2^ = 0.31, *p* = 0.1, Fig. 6). A similar, non-significant pattern was also noted for mite OTUs (perMANOVA *R*^2^ = 0.44, *p* = 0.1). Regardless of sampling protocol, compositional differences between the two forest sites (OF1, OF2) were consistently less than those between the forest sites and the meadow (OM1).

### Diversity patterns of soil arthropod assemblages

The 700K post-filtered reads from the 19 soil samples included 1,090 identified OTUs representing 123 families and 57 orders (Table 1). Most of these OTUs derived from non-arthropod invertebrates (449) followed by arthropods (356), protists (262), fungi (17), bacteria (8), and chordates (1). Although slopes varied among the samples, the accumulation curves for all identified OTUs did not reach an asymptote for any sample based on the eight technical replicates. A similar pattern was observed for the arthropods alone (Fig. 4). On average, 86 (SD = 42) OTUs including 25 (SD = 21) arthropod and 11 (SD = 12) mite OTUs were recovered from a sample.

**Figure 7:**
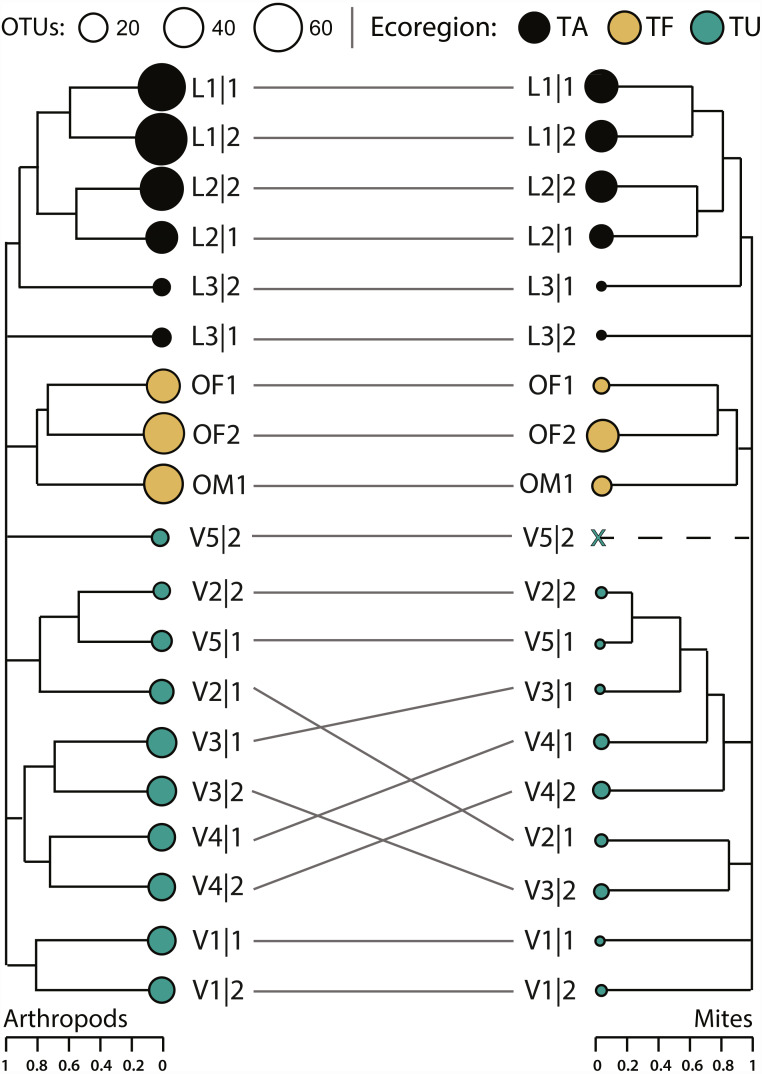
Hierarchical clustering of pairwise Sorensen dissimilarity values for arthropod and mite OTUs detected from 19 soil samples from 11 sites and three ecoregions. (TA, Taiga/Arctic Cordillera transition; TF, Temperate Forest; TU, Tundra) within Canada.

Compositional differences between samples increased significantly with geographic distance for both the arthropod (Mantel statistic *r* = 0.51, *p* = 0.001) and mite (Mantel statistic *r* = 0.41, *p* = 0.001) assemblages. The arthropod composition was significantly different between sites and between ecoregions, but differences among sites (perMANOVA *R* ^2^ = 0.44, *p* = 0.007) explained more variation in *β*-diversity than differences among ecoregions (perMANOVA *R*^2^ = 0.25, *p* < 0.001). However, hierarchical clustering of pairwise *β*-diversities revealed that most samples clustered by ecoregion while paired samples from just five sites (L1, L2, V1, V3, V4) formed sister groups (Fig. 6). More variation in mite *β*-diversity was also explained by differences among sites (perMANOVA *R*^2^ = 0.42, *p* = 0.049) than among ecoregions (perMANOVA *R*^2^ = 0.28, *p* < 0.01). The hierarchical clustering of pairwise mite *β*-diversities also revealed that most samples clustered by ecoregion, but three samples were outliers (L3—2, V1—1, V1—2). No mites were detected in V5—2, and samples from just two sites (L1, L2) formed sister groups (Fig. 7).

Hierarchical partitions of diversity were similar for arthropods and mites; *β*-diversity at the finest scale (sample) was low and increased with each hierarchical level with ecoregions contributing the greatest proportion of *β*-diversity to *γ*-diversity (Fig. 7). Most partitions were greater for all arthropods than for mites alone, but multiplicative partitions of *β*Site and *β*Ecoregion were higher for mites (Fig. 7). Compared to null models, partitions of *α*Sample were higher than expected by chance (*p* < 0.0001) for all arthropods (3.9%) and for mites (5.3%). Partitions of *β*-diversity were also higher than expected (*p* < 0.0001) for arthropods (additive = 12.3%, multiplicative = 20.8%) and mites (additive = 10.7%, multiplicative = 18.9%) at the ecoregion level, but not at the sample or site levels (Fig. S2).

## Discussion

The present study compared the efficacy of two bulk sampling approaches (specimens, soil) for the assessment of soil arthropod diversity by DNA metabarcoding. Interpretation of the resulting sequence data was constrained because just 41% of reads and 15% of OTUs were identified at any rank. Despite this constraint, a broad range of invertebrate and microbial taxa were detected from the analysis of just three specimen and 19 soil samples. The overall data revealed 410 arthropod OTUs belonging to 112 families, 25 orders, and nine classes. The level of taxonomic resolution differed among groups reflecting variation in the completeness of their reference library on BOLD. Resolution was typically highest for arthropods owing to extensive surveys of the Canadian fauna (de Waard et al., 2019; Hebert et al., 2016), including studies near ***rare*** (Telfer et al., 2015) and near Cambridge Bay (Pentinsaari et al., 2020). Identification success was highest for insects (Hebert et al., 2016) and spiders (Blagoev et al., 2016). By comparison, identification success for mites was low despite an extensive survey of the Canada fauna, confirming the prediction that many species in this group await barcode characterization (Young et al., 2019). Success was also low for highly diverse non-arthropod groups that are rarely surveyed in soil such as the bdelloid rotifers (Robeson et al., 2011) and protists, especially heterokonts (Pawlowski et al., 2012). Proteobacteria were likely also hyperdiverse but largely undetected due to a lack of close matches (*e.g.*, >85% similarity) in the reference library. Expanding reference library coverage for these groups would substantially increase the proportion of identified OTUs, raising the taxonomic resolution of soil metabarcoding studies. Despite biased and incomplete library coverage, our analyses demonstrate that DNA metabarcoding of bulk specimen and soil samples recovered many of the arthropod taxa isolated by Berlese-Tullgren funnels and revealed generally congruent patterns of *α*- and *β*-diversity.

### Taxon recovery

Bulk soil analysis recovered significantly more orders, families, and OTUs than specimen analysis reflecting the higher recovery of non-target taxa from bulk soil DNA. The amplification of non-target taxa from soil DNA extracts is inescapable with degenerate primers (Dopheide et al., 2019a; Marquina et al., 2019; Saitoh et al., 2016) like those employed in this study (Prosser et al., 2016). Degenerate primers will also amplify NUMTs, a problem that can be reduced by discarding OTUs with stop codons, or those lacking a close match in the reference library (Creedy et al., 2021). However, such filtering would also reduce the sensitivity of DNA metabarcoding studies generated by platforms prone to indel errors and from highly diverse assemblages that are poorly represented in the reference library. Despite our adoption of a modest quality filter, just one apparent NUMT was detected in our post-filtered dataset. However, discriminating NUMTs from sequencing artefacts is difficult, especially for those lacking stop codons or large indels. For example, nearly 20% of the OTUs in our dataset possessed a single bp indel in a homopolymer tract consistent with IonTorrent’s sequencing error profile (Piñol et al., 2015), but similar indels were observed in many suspected NUMTs from the positive control. Ultimately, degenerate primers are necessary to minimize amplification bias and to characterize a breadth of taxa as wide as the soil arthropods (Krehenwinkel et al., 2017) but the recovery of NUMTs will complicate data interpretation.

While most (59%) false positives encountered in the positive control were likely NUMTs, almost a third (31%) potentially derived from gut contents or other trace DNA associated with the specimens. The two unconfirmed families (Trombidiformes: Scutacaridae, Tydeidae) detected by bulk specimen analysis may also represent trace DNA but were most likely present in the sample and lost during voucher recovery, because of their small body size (<200 µm). Some tag-switching was observed in the negative controls although all cases had low abundances (<6 reads). As a result, abundance-based filtering was applied to the remaining samples but a few cases of tag-switching persisted in the positive control despite this intervention and may have also been present in the specimen/soil samples. In the positive control, tag-switching occurrences always represented less than 0.05% of the total read count for an OTU. As a consequence, they could have been eliminated by the use of dynamic abundance filtering based on total OTU abundance (in addition to the sample abundance-based filtering that was employed).

Despite the prevalence of non-target recovery, most (80%) of the arthropod families present in the specimen samples were recovered by metabarcode analysis. However, the present analysis undoubtedly recovered just a fraction of the species in each sample since sequences were recovered from less than a quarter of the BINs in the positive control in each run. These undetected taxa included species overlooked by the relatively low sequencing depth employed in this study because of their low biomass (Elbrecht, Peinert & Leese, 2017) or low affinity with the primers (Elbrecht et al., 2019). A previous analysis utilizing the same primer pair and sequencing platform as this study revealed that 100K reads were necessary to recover 95% of the species in a bulk sample that represented a simple challenge as it included a single individual of 374 arthropod species (Braukmann et al., 2019). Because the mean sequencing depth in our study averaged just 21K reads per technical replicate (169K per sample), sequencing coverage was clearly inadequate to reveal all taxa, as demonstrated by the fact that accumulation curves for OTUs across the 8 technical replicates reached an asymptote for just one of the 22 samples. The slopes of these curves were consistently higher for bulk soil than specimen samples, indicating that a much larger proportion of bulk soil richness was undetected. While optimizing extraction and PCR protocols will certainly improve OTU recovery from bulk soil samples (Dopheide et al., 2019b), future work should also evaluate the ideal level of replication and sequence coverage required to adequately capture bulk soil diversity (Lanzén et al., 2017). However, it is near certain that a 25x–50x increase in read depth will be required to reach asymptotic richness of a sample.

Although the composition of arthropod OTUs recovered by the paired specimen and soil samples were not statistically different, this result likely reflects a Type II error due to low sample size (*n* = 3) since a large proportion of the variation in *β*-diversity (*R*^2^ <0.3) was explained by compositional differences between the two sampling methods. While improving the recovery of OTUs may reduce compositional differences, they will more likely be magnified since the slopes of the accumulation curves suggest that OTU richness from bulk soil will greatly exceed that from specimens given sufficient sampling. Some compositional differences between the specimen and soil samples undoubtedly reflect the physical presence/absence of taxa in each subsample. However, most differences likely stem from systematic biases between the methods, since some taxa are infrequently isolated by Berlese-Tullgren funnels (André, Ducarme & Lebrun, 2002) while bulk soil will also detect rare or transient taxa through environmental traces of their DNA (Deiner et al., 2017). Relic DNA is also abundant in soils and may inflate estimates of arthropod richness (Nagler et al., 2018). However, the magnitude of this effect depends on the persistence of relic DNA in the environment (Nielsen et al., 2007) which remains uncertain for arthropods in the soil. Regardless of the cause, it is clear that bulk specimen and soil analyses capture complementary portions of the soil fauna.

### Patterns of arthropod *α*- and *β*-diversity

Despite low read depth, limited sample size, and compositional differences, results from the three paired specimen and soil samples indicated similar patterns of arthropod *α*- and *β*-diversity. These results confirm prior studies that revealed similar estimates of arthropod order, family, and OTU richness obtained from metabarcode analyses of soil and soil-isolated specimens (Dopheide et al., 2019a; Oliverio et al., 2018), demonstrating their ability to recover ecological signal despite the incomplete recovery of OTUs. For example, compositional differences between the meadow and forested sites were consistently greater than differences between the pair of forested sites regardless of the taxon or protocol examined. These results support prior studies revealing strong differentiation between grassland and forest soil communities through morphological (Caruso, Taormina & Migliorini, 2012) and molecular analyses (Arribas et al., 2021). Diversity patterns revealed by easily collected soil samples may parallel those seen in above-ground communities (*i.e.*, leaf litter and Malaise trap samples; Yang et al., 2014). If so, bulk soil analyses could provide a rapid, cost-effective solution for terrestrial biomonitoring. Although sufficient replication and read depth will be necessary to develop comprehensive baselines for the soil fauna using this method, adopting standardized methods should also limit analytical biases and allow robust assessments of diversity patterns despite undersampling (Mathieu et al., 2020).

Our comparison of soil samples across the three ecoregions confirmed previously observed patterns of distance-decay congruent with dispersal-limited, neutral community assembly for mites and other soil arthropod assemblages (Arribas et al., 2021; Young et al., 2019; Zinger et al., 2019). The importance of neutral processes in shaping soil arthropod assembly was supported by partitions of *β*-diversity at the sample and site scales which did not differ from null models. However, high turnover was observed even between close sites (*e.g.*, between transects just 10 m apart). This pattern could result from undersampling (Beck, Holloway & Schwanghart, 2013), but may also reflect the highly patchy distributions of soil arthropods (Bardgett, 2002). For example, environmental factors such as soil type and ground cover have been linked to high variability in local soil mite assemblages (Lindo & Winchester, 2009; Meehan, Song & Proctor, 2018), while moisture, temperature, and organic matter are known to shape the assembly of soil arthropod communities at very fine scales (Ghiglieno et al., 2020). Environmentally driven assembly at a micro scale was further supported by higher than expected partitions of *α*Sample, indicating that mechanisms promoting species coexistence support locally rich assemblages of soil arthropods (Leibold & Chase, 2018). Partitions of *β* Ecoregion were also higher than expected by chance, and samples typically clustered by ecoregion, mirroring patterns of non-neutral assembly previously observed for soil mite assemblages in Canadian protected areas (Young et al., 2019). Most communities are structured by both neutral and niche processes (Cottenie, 2005), and our results affirm the importance of both in shaping soil arthropod diversity across the Canadian landscape.

## Conclusion

This study has demonstrated that DNA metabarcoding can greatly extend knowledge of soil arthropod communities. Although there was little species overlap, patterns of *α*- and *β*-diversity for soil arthropods were congruent between bulk soil and specimen samples. Because of its simplicity, the analysis of soil samples is conducive to large-scale surveys. Increased sequencing depth will improve taxon recovery, while expansion of the DNA barcode reference library will enhance the resolution of taxonomic assignments generated from metabarcode data.

##  Supplemental Information

10.7717/peerj.12845/supp-1 Supplemental Information 1 Collection details for 22 soil samples collected from 11 sites across Canada including three used for specimen and 19 for soil DNA metabarcode analysis

10.7717/peerj.12845/supp-2 Supplemental Information 2 Summary of laboratory samples for the eight extraction replicates from each of 22 field samples and six laboratory controls

10.7717/peerj.12845/supp-3 Supplemental Information 3 OTU read abundance table for the eight extraction replicates from each of 22 field samples and six laboratory controls

10.7717/peerj.12845/supp-4 Supplemental Information 4 OTU read abundance table including eight extraction replicates from each of the 22 field samples with the maximum read abundance in negative controls subtracted from all other occurrences of that OTU

10.7717/peerj.12845/supp-5 Supplemental Information 5 Summary of morphologically identified specimens recovered from three DNA metabarcoded specimen samples following DNA extraction

10.7717/peerj.12845/supp-6 Supplemental Information 6 The number of OTUs recovered for all taxa, arthropods, and mites from three paired samples of specimens and soil analysed by DNA metabarcoding Numbers within each circle represent the OTUs unique to a protocol, while those within the zones of overlap were detected by both protocols.

10.7717/peerj.12845/supp-7 Supplemental Information 7 Additive and multiplicative hierarchical partitioning of Sorensen dissimilarity for arthropod and mite OTUs detected from 19 soil samples showing the average *β*-diversity contributed by each spatial scale (Sample, Site, Ecoregion) Partitions which are significantly different than expected by chance are denoted by an asterisk (*).

10.7717/peerj.12845/supp-8 Supplemental Information 8 R scripts for bioinformatics and statistical analyses

## Additional Information and Declarations

### Field Study Permissions

The following information was supplied relating to field study approvals (i.e., approving body and any reference numbers): Samples were collected with permission at the ***rare*** Charitable Research Reserve. Samples collected in the vicinity of the Canadian High Arctic Research Station were contained with the Department of Environment Wildlife Research Permit (WL 2018-044). A permit was also obtained through the Canada C3 Initiative for sampling in National Parks in Labrador (ANP-2017-25145) and collecting in other sites was approved by the Nunatsiavut Government Research Advisory Committee.

### Data Availability

The following information was supplied regarding data availability: The raw sequence data is available at NCBI’s Sequence Read Archive (BioProject: PRJNA755277). The R scripts for bioinformatics and statistical analysis are available in the Supplementary Files.
