# Supplementary figures and images for: Unearthing soil arthropod diversity through DNA metabarcoding
Source: PeerJ. 2022 Feb 1;10:e12845. doi: 10.7717/peerj.12845 (PMC8815377; doi:10.7717/peerj.12845)

**OF1**

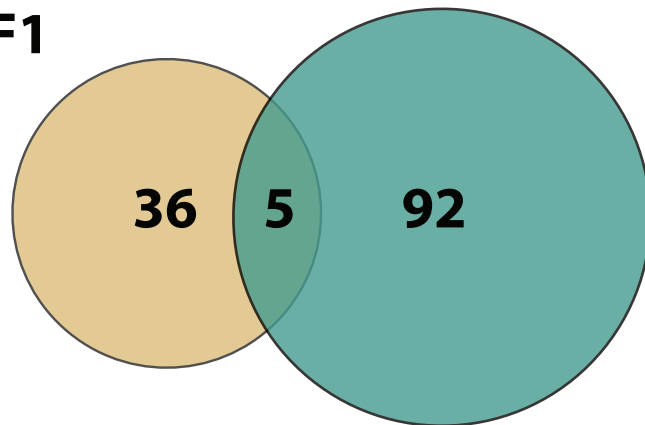

**OF2**

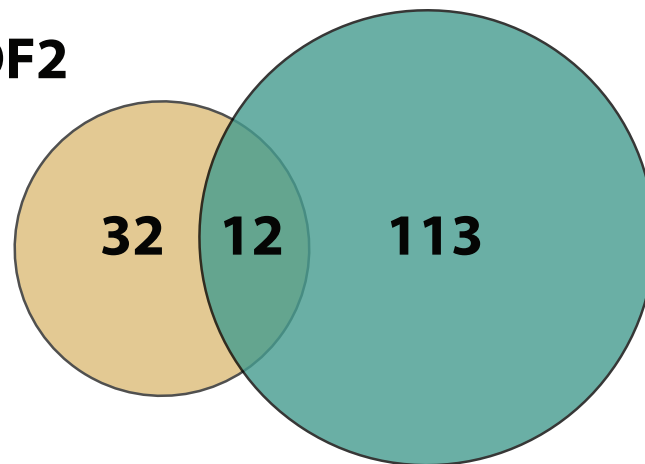

**OM1**

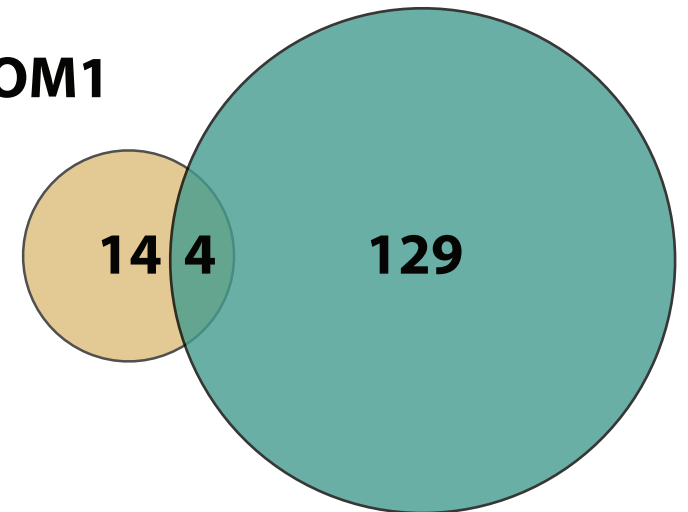

**All taxa**

---

**OF1**

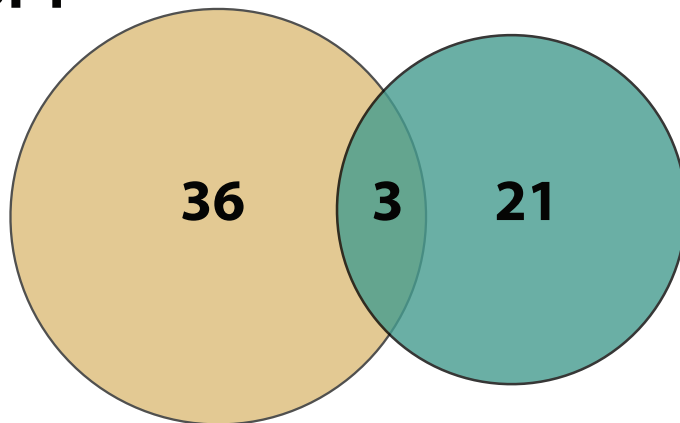

**OF2**

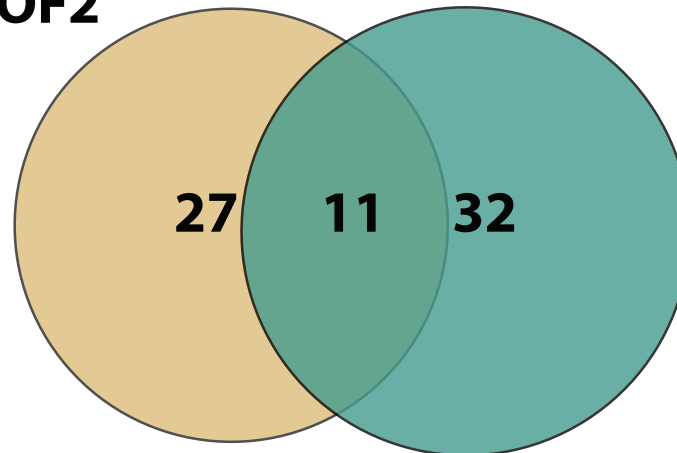

**OM1**

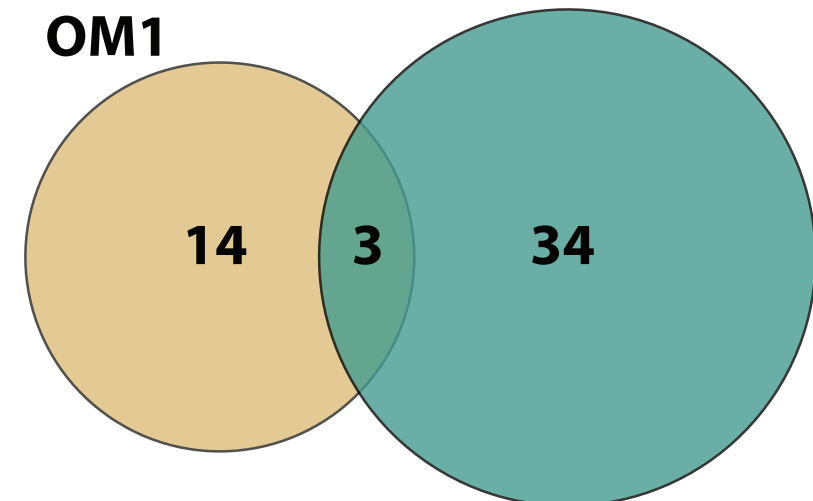

**Arthropods**

---

**OF1**

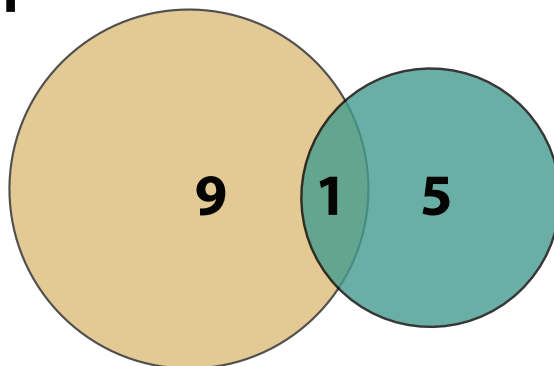

**OF2**

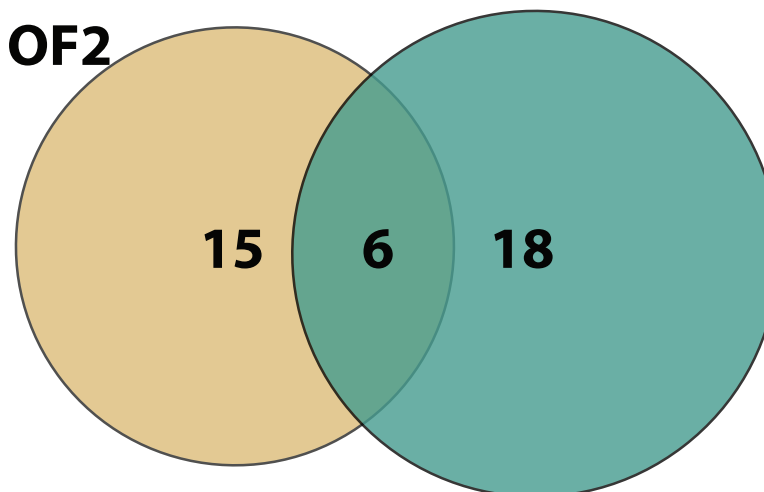

**OM1**

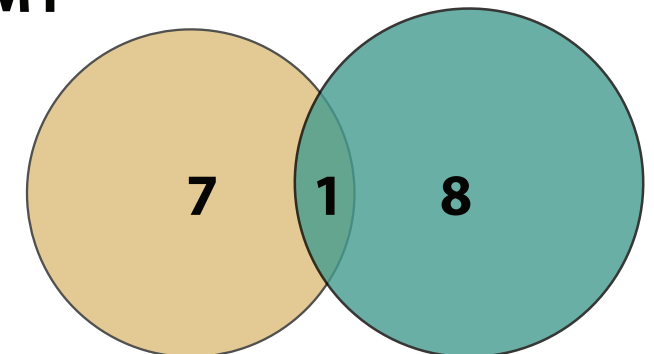

**Mites**

Protocol: 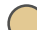 Specimens 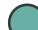 Soil

Supplement: Supplemental Information 6 — Numbers within each circle represent the OTUs unique to a protocol, while those within the zones of overlap were detected by both protocols. [file peerj-10-12845-s006.pdf]

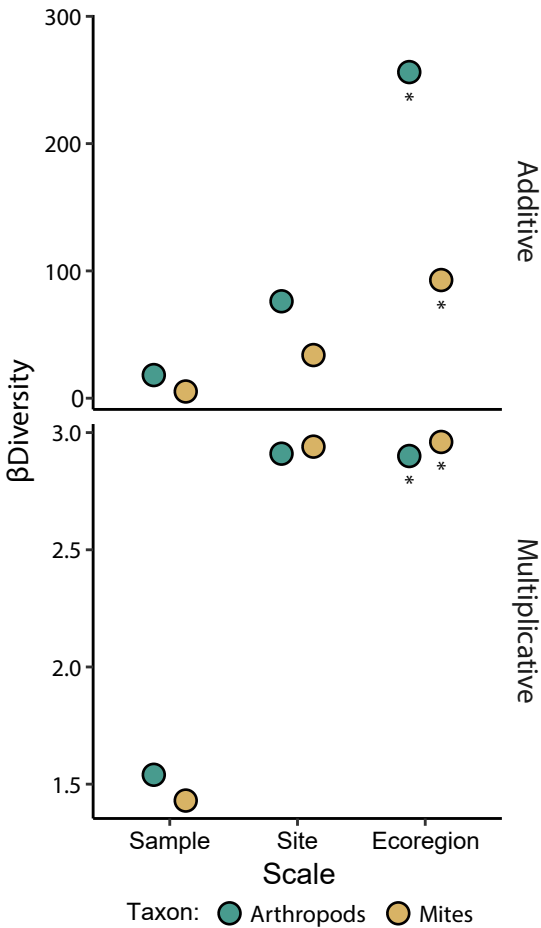

Supplement: Supplemental Information 7 — Partitions which are significantly different than expected by chance are denoted by an asterisk (*). [file peerj-10-12845-s007.pdf]
